# Statin Use Is Not Associated with Improved Progression Free Survival in Cetuximab Treated *KRAS* Mutant Metastatic Colorectal Cancer Patients: Results from the CAIRO2 Study

**DOI:** 10.1371/journal.pone.0112201

**Published:** 2014-11-06

**Authors:** Lisanne L. Krens, Lieke H. J. Simkens, Jara M. Baas, Els R. Koomen, Hans Gelderblom, Cornelis J. A. Punt, Henk-Jan Guchelaar

**Affiliations:** 1 Dept. of Clinical Pharmacy and Toxicology, Leiden University Medical Center, Leiden, The Netherlands; 2 Dept. of Medical Oncology, Academic Medical Center, University of Amsterdam, Amsterdam, The Netherlands; 3 Dept. of Clinical Oncology, Leiden University Medical Center, Leiden, The Netherlands; Queen Mary Hospital, Hong Kong

## Abstract

Statins may inhibit the expression of the mutant KRAS phenotype by preventing the prenylation and thus the activation of the KRAS protein. This study was aimed at retrospectively evaluating the effect of statin use on outcome in *KRAS* mutant metastatic colorectal cancer patients (mCRC) treated with cetuximab. Treatment data were obtained from patients who were treated with capecitabine, oxaliplatin bevacizumab ± cetuximab in the phase III CAIRO2 study. A total of 529 patients were included in this study, of whom 78 patients were on statin therapy. In patients with a KRAS wild type tumor (n = 321) the median PFS was 10.3 vs. 11.4 months for non-users compared to statin users and in patients with a KRAS mutant tumor (n = 208) this was 7.6 vs. 6.2 months, respectively. The hazard ratio (HR) for PFS for statin users was 1.12 (95% confidence interval 0.78–1.61) and was not influenced by treatment arm, KRAS mutation status or the KRAS*statin interaction. Statin use adjusted for covariates was not associated with increased PFS (HR = 1.01, 95% confidence interval 0.71–1.54). In patients with a KRAS wild type tumor the median OS for non-users compared to statin users was 22.4 vs. 19.8 months and in the KRAS mutant tumor group the OS was 18.1 vs. 14.5 months. OS was significantly shorter in statin users versus non-users (HR = 1.54; 95% confidence interval 1.06–2.22). However, statin use, adjusted for covariates was not associated with increased OS (HR = 1.41, 95% confidence interval 0.95–2.10). In conclusion, the use of statins at time of diagnosis was not associated with an improved PFS in *KRAS* mutant mCRC patients treated with chemotherapy and bevacizumab plus cetuximab.

## Introduction

**Figure 1:**
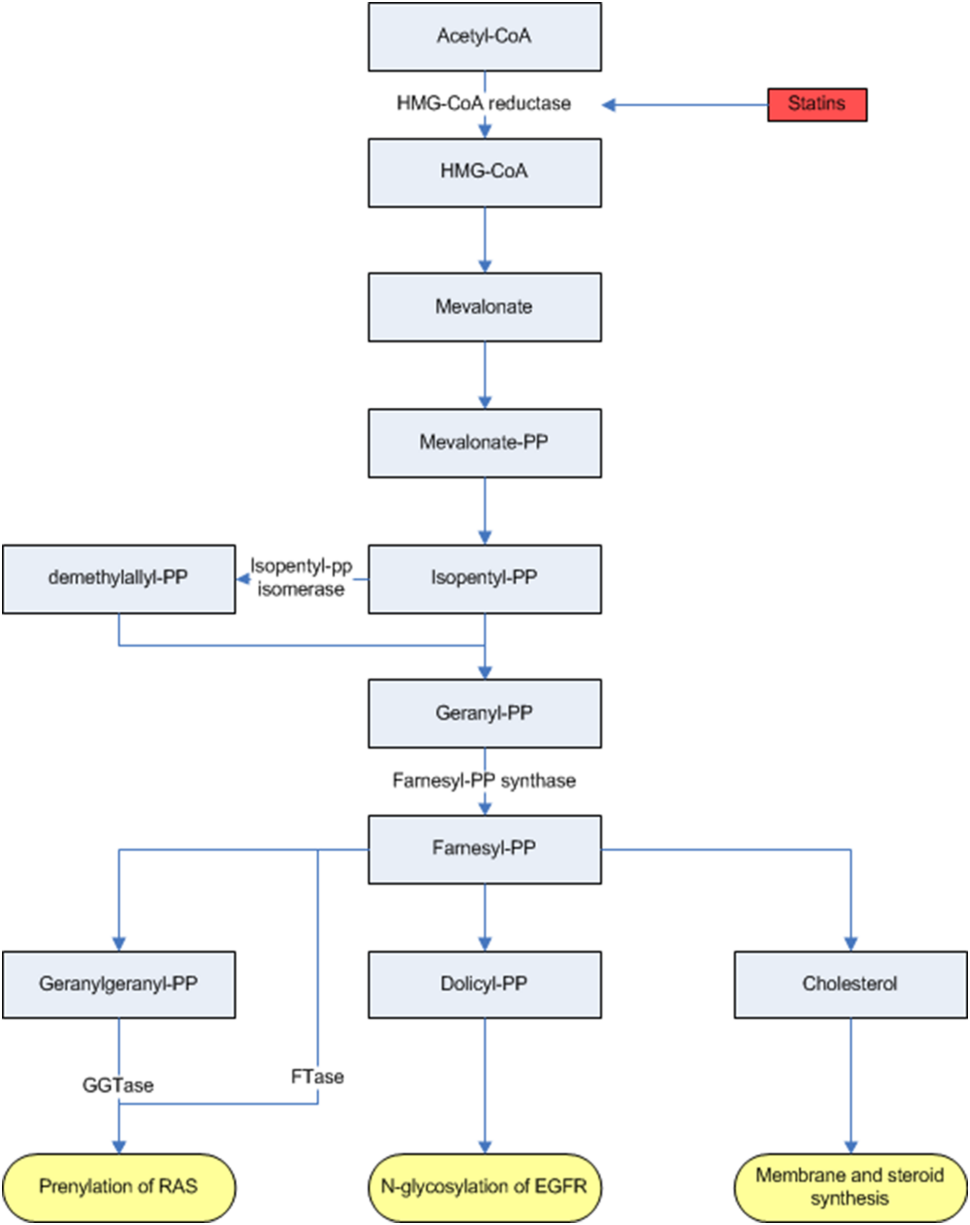
Overview of the mevalonate pathway and the inhibition of HMG-CoA by statins. *Mevalonate pathway causes prenylation of ras, N-glycosylation of EGFR and membrane and steroidsynthesis. Statins have inhibitory effects on the mevalonate pathway and thus on prenylation of k-ras. Abbreviations: Acetyl-CoA, Acetyl coenzyme A; EGFR, epidermal growth factor receptor; FTase, farnesyltransferase; GTase, geranylgeranyltransferase; HMG-CoA (reductase), 3-hydroxy-3-methyl-glutaryl-CoA reductase; -PP, -pyrophosphate.*

Statins are widely prescribed to lower blood cholesterol concentration and have shown to reduce the risk of cardiovascular events and mortality [1]. In addition, the use of statins have been associated with a reduced risk of malignancies in a variety of organ sites, such as colon, rectum, lungs and liver [2]. Statins inhibit cholesterol synthesis via inhibition of the mevalonate pathway but also lower protein prenylation (Figure 1). As a posttranscriptional process, protein prenylation is crucial for several cancer cell growth related proteins, such as KRAS. The KRAS protein is activated by post-translational prenylation by binding farnesyl (C15) and geranylgeranyl (C17) moieties, both products of the mevalonate pathway. After prenylation the KRAS protein becomes lipophilic and translocates to the cellular membrane to exerts its function [3].

Epidermal Growth Factor Receptor (EGFR) inhibitors, such as cetuximab and panitumumab, have shown survival benefit in combination with chemotherapy and as monotherapy in metastatic colorectal cancer (mCRC) patients [4]. Their benefit is restricted to patients with a *KRAS* exon 2 wild type tumor [5], which recently was further narrowed to *RAS* wildtype exon 2–4 tumors [6]. In patients with a *KRAS* mutated tumor, the RAS pathway is permanently activated, leading to constant cell signalling and proliferation independent of the EGFR.

Statins may inhibit the expression of the mutant KRAS phenotype by preventing the prenylation of the KRAS protein and normalize the phenotype into KRAS wild type and therefore render *KRAS* mutant colorectal cancers sensitive to EGFR antibodies [7]. We hypothesize that *KRAS* mutant cetuximab treated CRC patients with concurrent statin use have a favourable outcome from EGFR therapy compared to non-users. This study was aimed at retrospectively evaluating the effect of statin use in *KRAS* mutant mCRC patients treated with cetuximab.

## Materials and Methods

### Patients

For this analysis prospectively collected data were obtained from mCRC patients participating in the CAIRO2 study of the Dutch Colorectal Cancer Group (DCCG). Patients were randomised between capecitabine plus oxaliplatin (CAPOX) and bevacizumab, study arm A, and the same regimen plus cetuximab, study arm B (ClinicalTrials.gov NCT00208546 [8]). Cetuximab was administered at a dose of 400 mg/m^2^ on the first day followed by 250 mg/m^2^ weekly thereafter. Details of eligibility criteria and results have been reported elsewhere [8] Patients with an tumor with an unknown *KRAS* mutation status were excluded from this analysis.

### Drug exposure

Statin use was defined as the use of a statin at visit 0, the randomisation or at visit 1, 3 weeks after start of treatment. All statins (ATC-codes C10AAXX), commercially available in The Netherlands within the study period were included: simvastatin, pravastatin, atorvastatin, rosuvastatin and fluvastatin.

### Potential confounders

Use of drugs related to progression and development of colorectal carcinoma such as non-steroidal anti-inflammatory drugs (NSAID’s), aspirin, fibrates and bisphosphonates at visit 0 or 1 were considered as potential confounders. The use of these drugs was recorded. If the use of these drugs in the study population was less then <1%, the drug was excluded from the further analysis. The use of fibrates was excluded, from the analysis because of the low prevalence (<1%).

### Outcome measures

The primary outcome measure in this study was to assess the influence of statin use during chemotherapy with CAPOX-bevacizumab and cetuximab on progression free survival (PFS) in patients with *KRAS* mutant CRC. Furthermore, we examined the influence on overall survival (OS).

### Statistical analysis

Baseline characteristics were compared between statin users and nonusers using a χ^2^ test for categorical comparisons and for continuous variables the Student’s t-test was used.

PFS was calculated as time from randomisation to the first documented progression, death or last follow up, whichever came first. OS was calculated as time from randomisation to death or last follow up. Kaplan-Meier survival estimates were calculated to determine the effect of statin use on PFS and OS in the cetuximab treated group by stratifying the study population into two groups according to *KRAS* status. For comparison between the statin users and non-users a log-rank test was used.

Cox proportional hazard models were used to determine whether the statin use in patients with *KRAS* mutant tumors treated with cetuximab was a significant predictor of PFS and OS. Instead of a subgroup analysis based on KRAS status and treatment arm, we used a Cox proportional hazard model, to study the effects of statins in cetuximab treated patients and compare it to non-cetuximab users to exclude a general statin effect. The following parameters were used in the model, statin use, *KRAS* mutation status, treatment arm, allowing for a different effect of statins between *KRAS* mutant and wildtype tumors by means of an effect modifier in the model. In the multivariate analysis we included potential confounders with a p-value of <0.10 from the baseline univariate analysis, between statin user and non-users.

The deviating baseline characteristics between statin users and non-users with a p-value of <0.1 were also included in the multivariate analysis, e.g. prior adjuvant therapy, number of affected organs, and age.

The data are expressed as hazard ratios (HR), 95% CI intervals and P values. All statistical tests were two sided and p values <0.05 were considered statistically significant unless stated otherwise. All statistical analyses were performed using SPSS version 20 (SPSS for Windows, SPSS Inc., Chicago, IL, USA).

## Results

### Baseline patient characteristics according to statin use

795 patients were enrolled in the CAIRO2 study. A total of 529 patients from the CAIRO2 study were included in this analysis, 266 patients were excluded based on unknown *KRAS* mutation status, due to retrospective genotyping of the *KRAS* mutation status of the tumor, because the CAIRO2 study was performed in the pre KRAS era. A total of 78 patients were on statin therapy, of whom 43 patients were classified in treatment group A CAPOX-B and 35 in group B, CAPOX-B with cetuximab. 451 patients did not use a statin, of whom 225 patients were in group A and 226 to group B. The study population is described in Table 1. It is noteworthy that patients in the statin group were older (67.1 vs. 61.9 p<0.001), more likely to be an aspirin user (44.9% vs. 6.4% p<0.001) and had a lower number of affected organs (>1 organ: 48.7% vs. 60.3% p = 0.049) compared to patients who were not on statins. These deviating baseline characteristics between statin users and non-users with a p-value of <0.1 were included in the multivariate analysis.

**Table 1:** Patient Characteristics.

Parameter	Statin users	Non-statin users	P value
	N (%)	N (%)	
**Patients**			
total	78 (14.0)	451 (86.0)	
**KRAS status**			0.112
Wildtype	41 (52.6)	280 (62.1)	
Mutant	37 (47.4)	171 (37.9)	
**Sex**			0.269
Male	50 (64.1)	259 (57.4)	
female	28 (35.9)	192 (42.6)	
**Arm**			0.393
CAPOX-B	43 (55.1)	225 (49.9)	
CAPOX-B + cetuximab	35 (44.9)	226 (50.1)	
**Serum LDH**			0.624
Normal	48 (61.5)	288 (63.9)	
Above normal	30 (38.5)	159 (35.3)	
**WHO performance status**			0.467
0	29 (62.8)	306 (67.8)	
1	28 (35.9)	145 (32.2)	
**Prior adjuvant therapy**			0.055
No	70 (89.7)	364 (80.7)	
Yes	8 (10.3)	87 (19.3)	
**Number of affected organs**			0.049
1 organ	40 (51.3)	177 (39.2)	
>1 organ	38 (48.7)	272 (60.3)	
**Site of primary tumor**			0.871
Colon	34 (43.6)	209 (46.3)	
Rectum	19 (24.4)	115 (25.5)	
Recto sigmoid	25 (32.1)	126 (27.9)	
**Age**			<0.001
Mean	67.1	61.9	
Range	46.1–83.6	27.6–80.0	
**Statin**			
Pravastatin	13 (16.7)		
Simvastatin	28 (35.9)		
Atorvastatine	23 (29.5)		
Rosuvastatin	11 (14.1)		
Fluvastatin	3 (3.8)		
**NSAID user**	6 (7.7)	45 (10.0)	0.528
**Aspirin user**	35 (44.9)	29 (6.4)	<0.001
**Bisphophonate user**	2 (2.6)	5 (1.1)	0.299
**Fibrate user**	1 (1.3)	1 (0.2)	-

### Effect of statin use on progression free survival

**Figure 2:**
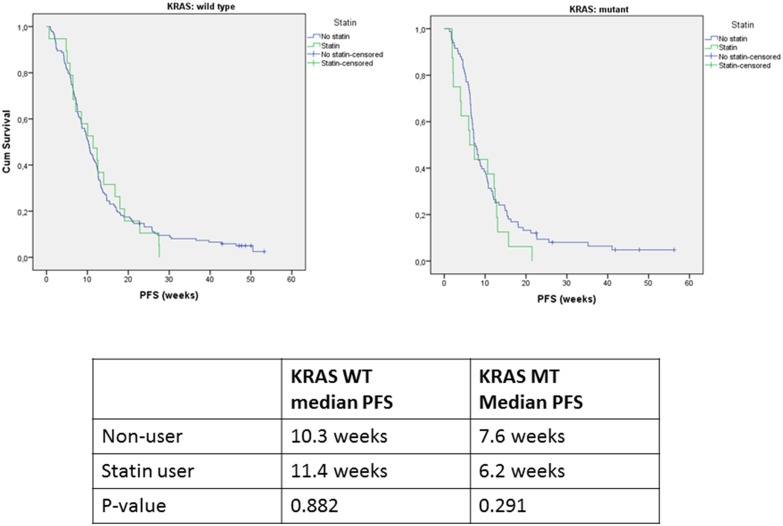
Kaplan-Meier plots for progression free survival for patients with KRAS wild type (19 statin-users and 145 nonusers) and KRAS mutant (16 statin-users and 83 nonusers) tumors treated with capecitabine, oxaliplatin, bevacizumab and cetuximab.

Statin use alone did not have a statistically significant effect on PFS of cetuximab treated patients with a *KRAS* mutant tumor (Figure 2). In patients with a *KRAS* wild type tumor, the median PFS was 10.3 vs. 11.4 months (p = 0.882) for nonusers compared to statin users, and in the *KRAS* mutant group 7.6 vs. 6.2 months (p = 0.291), respectively. The hazard ratio (HR) of PFS was 1.12 (95% confidence interval 0.78–1.61) and was not influenced by treatment arm, *KRAS* mutation status or the KRAS*statin interaction.

In the multivariate analysis, the covariate adjusted HR for PFS was 1.01 (95% CI 0.71–1.54) for statin users.

### Effect of statin use on overall survival

**Figure 3:**
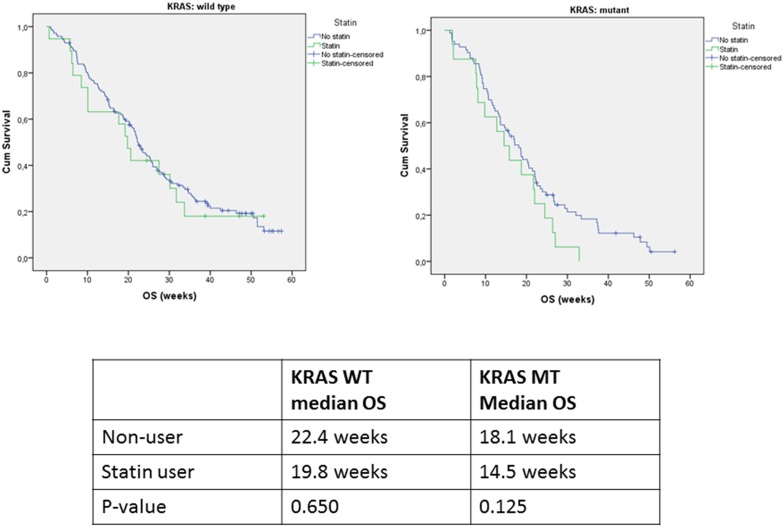
Kaplan-Meier plots for overall survival for patients with KRAS wild type (19 statin-users and 145 nonusers) and KRAS mutant tumors (16 statin-users and 83 nonusers) treated with capecitabine, oxaliplatin, bevacizumab and cetuximab.

Among patients with a *KRAS* wild type tumor, the median OS for non-users compared to statin users was 22.4 vs. 19.8 months (p = 0.650), in patients with a *KRAS* mutant tumor the median OS was 18.1 vs. 14.5 months (p = 0.125) (Figure 3), respectively. The OS was different between statin users and non-users (HR = 1.54 for statin users 95% confidence interval 1.06–2.22) in the crude analysis. However, the covariate adjusted hazard ratio for OS was not associated with increased survival in the statin users (HR = 1.41 for statin users, 95% confidence interval 0.95–2.10).

## Discussion

The results of this cohort study of patients diagnosed with metastatic CRC show that the use of statins is not associated with an improved PFS in patients with *KRAS* mutant tumors treated with cetuximab.

To the best of our knowledge, this is the first study investigating the effects of statin use on outcome in metastatic CRC patients in relation to *KRAS* mutation status and use of cetuximab. Preclinical studies have shown the antitumor effect of statins in CRC by a variety of mechanisms on cell proliferation. The leading hypothesized mechanism of statins is the inhibition of farnesylation of the KRAS protein [7], [9]. We hypothesized that *KRAS* mutant CRC treated with cetuximab benefit from statin use, because statins may phenoconvert the overactive KRAS protein to a more wildtype KRAS phenotype and thereby render these tumors sensitive to cetuximab treatment. Instead of stratifying for *KRAS* status and treatment arm and performing a subgroup analysis, a Cox proportional hazard model in the complete cohort of 529 patients was used, allowing for a different effect of statins between KRAS mutant and wildtype tumors by means of an effect modifier. This study design allows to exclude a possible “generic” effect of statins on survival, because patients with a *KRAS* wildtype tumor and patients in the arm without cetuximab were also included in the analysis. We did not observe an effect of statin use on the wildtype *KRAS* tumors and thus no effect on cetuximab sensitivity. Moreover, we found no association between statin use and progression-free or overall survival in patients with a mutant *KRAS* tumor and therefore our study results do not support our hypothesis.

A possible explanation for the lack of effect of statins is that the cohort existed of patients with CRC with metastatic disease and hence a relatively short progression-free survival to demonstrate a modulating effect of statins on the efficacy of cetuximab. Secondly, in preclinical studies high doses of statins are used to treat cancers, aiming at inducing a cytotoxic treatment effect. The high concentrations used in those in vitro cell cultures are most likely not reached if the registered dose of statin for cardiovascular prevention is prescribed [10]. On the other hand, the registered doses decrease cholesterol levels and subsequently, the formation of prenylgroups, is reduced and as a consequence the prenylation of KRAS is inhibited [11].

This retrospective cohort study has some limitations. The included patients used different doses and statin types. We did not analyse the type, duration or dose of statin given, so we were unable to access the individual effect of these characteristics on the endpoints of the study. The bioavailability, pharmacokinetics and pharmacodynamics could be significantly different; however, we expect that patients were adequately treated for hypercholesterolemia as it is common practice to titrate patients based upon monitoring of their cholesterol levels. The proposed effect of the statins in this study is inhibition of formation of farnesyl- and geranylgeranylgroups which are essential for KRAS activation and also closely related to the main statin effect namely HMG CoA reductase inhibition. Therefore, all statins could be combined in this study. Since patients included in this study were on stable statin dose we assumed that target levels of cholesterol were reached. Consequently, this also implies that effective inhibition of formation of farnesyl- and geranylgeranylgroups was reached at the individualized statin dose. We thoroughly screened the patients’ co-medication to minimize the exposure misclassifications, nonetheless, the uncertainty of patients’ compliance to the prescribed regimen and the lack of prescription information may influence the study results. Patients with statin use at randomisation or first visit were included in the statin user group. We did neither record patients with prior statin use, nor new users after randomisation. Therefore, no cumulative statin dose could be calculated, which might be an important factor, because it gives more information about the potential dose relationships and causality. The effect of different statins was not studied, because of the limited number of patients per subgroup. Differences between statins may exist since the hydrophilic statins, rosuvastatin and atorvastatin, have a decreased ability to penetrate cell membranes [12].

Another important limitation of this study is that patients were treated with the combination of chemotherapy, bevacizumab and cetuximab. Hypertension, a common site effect of bevacizumab is correlated with a better survival in CRC patients treated with bevacizumab [13]. A possible negative interaction between bevacizumab and cetuximab may have caused less hypertension in the cetuximab treated group, which contributed to the negative outcome of the CAIRO2 study [8]. So, for this study it means that the outcomes in the cetuximab treated group may have been influenced by the negative interaction between bevacizumab and cetuximab.

Obviously, PFS may be confounded by many factors. However, in our study outcomes were controlled for the main potential drug confounders, NSAID’s, aspirin and bisphosphonates as well as for prior adjuvant therapy, number of organs effected and age. Nonetheless, confounding from unknown variables is still possible.

For testing a difference in effect on treatment between statin user and nonuser, PFS is the preferred primary endpoint. By studying PFS, a direct drug effect of statins on the cetuximab efficacy can be determined. A pronounced disadvantage of overall survival as an endpoint for this study is that this endpoint is less closely related to the drug effects. In the secondary analysis the use of statins in the unadjusted model was associated with a decrease of overall survival in the statin user group. A feasible explanation for the observed effect is that the statin users tend to be older and seemed to be less healthy, with a higher incidence of comorbidities then non-statin users, a confounding by indication. In the covariate adjusted cox regression this decrease of survival was not significant.

Due to the retrospective nature of this study we were not able to present data about KRAS prenylation levels of patient on statin therapy. In studies were the effects of statins are researched, data on prenylation levels of KRAS would be of great value, however at the moment a good assay to determine prenylation levels is lacking. To date, a number of studies have investigated statin use, CRC risk and clinical outcomes with inconclusive findings. Numerous studies and meta-analysis have investigated whether statin use reduces the risk of developing CRC [14], [15]. Fewer studies focus on effects of statin after diagnosis during treatment [16]–[21]. The study of Mace et al. [16] showed that rectal cancer patients in the statin cohort treated with neo-adjuvant chemoradiation had a better response (65.7% versus 48.7%, p = 0.004) and lower median regression rate (1 versus 2, p = 0.01). Two other studies [19], [20] in patients with rectal cancer treated with neo-adjuvant chemoradiation showed similar results indicating an association between statin use and response. However, in a study of Ng et al. [17], statin use during and after adjuvant chemotherapy among patients with stage 3 colon cancer was not associated with improved disease free survival, recurrent free survival or overall survival. In a prospective study of Lee et al. [18] the addition of simvastatin 40 mg, daily, to irinotecan, 5-fluorouracil, and leucovorin (FOLFORI) to first-line treatment in metastatic CRC patients showed promising antitumor activity and no additional adverse effects. These studies show that statin use in combination with systemic treatment for CRC may have some effect, but do not allow definite conclusions. However, all the above mentioned studies adressed the general cytotoxic effects of statins regardless of the *KRAS* status of the tumors. In our cohort we had the unique opportunity to study the effect of statins on cetuximab efficacy in CRC in relation to *KRAS* mutation status. Molecular data is warranted to study the exact mechanism of statins and their ability to potentiate chemotherapeutic agents. In new studies with statins, molecular data from tumors and patients should be collected, this data help to understand the involved mechanisms.

In conclusion, the use of statins at time of diagnosis was not associated with an improved PFS or OS in metastatic colorectal cancer patients with a *KRAS* mutant tumor treated with combination chemotherapy bevacizumab and cetuximab.
